# New insights into the genetic resistance to paratuberculosis in Holstein cattle via single-step genomic evaluation

**DOI:** 10.1186/s12711-022-00757-z

**Published:** 2022-10-15

**Authors:** Marie-Pierre Sanchez, Thierry Tribout, Sébastien Fritz, Raphaël Guatteo, Christine Fourichon, Laurent Schibler, Arnaud Delafosse, Didier Boichard

**Affiliations:** 1 Université Paris Saclay, INRAE, AgroParisTech, GABI, 78350 Jouy-en-Josas, France; 2 Eliance, 149 Rue de Bercy, 75012 Paris, France; 3 Oniris, INRAE, BIOEPAR, 44300 Nantes, France; 4 GDS Orne, 61000 Alençon, France

## Abstract

**Background:**

Bovine paratuberculosis, or Johne’s disease (JD), is a contagious and incurable disease caused by *Mycobacterium avium* subsp. *paratuberculosis* (MAP). It has adverse effects on animal welfare and is very difficult to control, leading to serious economic consequences. An important line of defense to this disease is host genetic resistance to MAP, which, when it will be more fully understood, could be improved through selective breeding. Using a large dataset of Holstein cows (161,253 animals including 56,766 cows with ELISA serological phenotypes and 12,431 animals with genotypes), we applied a single-step single nucleotide polymorphism (SNP) best linear unbiased prediction approach to investigate the genetic determinism underlying resistance to this disease (heritability estimate and identification of relevant genomic regions) and estimated genetic trends, reliability, and relative risk factors associated with genomic predictions.

**Results:**

Resistance to JD was moderately heritable (0.14) and 16 genomic regions were detected that accounted for at least 0.05% of the breeding values variance (GV) in resistance to JD, and were located on chromosomes 1, 3, 5, 6, 7, 19, 20, 21, 23, 25, and 27, with the highest percentage of variance explained by regions on chromosomes 23 (0.36% GV), 5 (0.22% GV), 1 (0.14% GV), and 3 (0.13% GV). When estimated for the whole chromosomes, the autosomes with the largest overall contributions were chromosomes 3 (5.3% GV), 10 (4.8%), 23 (4.7%), 1 (3.6%), 7 (3.4%), 5 (2.9%), 12 (2.5%), 11 (2.2%), and 13 (2%). We estimated a slightly favorable genetic trend in resistance to JD over the last two decades, which can be explained by a low positive genetic correlation between resistance to JD and total merit index (+ 0.06). Finally, in a validation population of 907 cows, relatively reliable genomic predictions (reliability = 0.55) were obtained, which allowed the identification of cows at high risk of infection.

**Conclusions:**

This study provides new insights into the genetic determinism of resistance to JD and shows that this trait can be predicted from SNP genotypes. It has led to the implementation of a single-step genomic evaluation that should rapidly become an effective tool for controlling paratuberculosis on French Holstein farms.

## Background

Paratuberculosis, or Johne’s disease (JD), is a mycobacterial disease caused by *Mycobacterium avium* subsp. *paratuberculosis* (MAP). Worldwide, JD is very common in livestock and primarily affects domestic ruminants [1]. In Europe, the cattle sector is particularly affected, with half of all herds being infected and the within-herd prevalence reaching 20% [2].

After being contaminated in utero or, more frequently, via the intake of MAP-infected feces as a young calf, animals experience a long latency phase that can last several years. Subclinical symptoms of JD are weight loss and reduced milk production, together with an inconsistent humoral immune response and fecal shedding. Such cases are difficult to detect and can contaminate their environment for years. The disease often evolves into chronic granulomatous enteritis and, in its clinical form, JD eventually results in chronic diarrhea and severe emaciation, ending in the death of the animal [3]. No effective treatment is available and vaccination is restricted because of the risk of interference with diagnostic methods used for bovine tuberculosis [4]. The most commonly used diagnostic tests are fecal culture, serum ELISA, milk ELISA, and fecal PCR, which are characterized by varying sensitivities and specificities depending on the stage of the infection.

As a result, JD is very difficult to control, which results in adverse effects on animal welfare and serious economic consequences [5]. Recent efforts to reduce the impact of this disease have focused on the role of host genetics in resistance to MAP. Heritability estimates for this trait range between 0.03 and 0.27 [6], and genome-wide association studies (GWAS), which were conducted using genotypes of single nucleotide polymorphisms (SNPs) at different densities (e.g. [7, 8]), have detected various genomic regions, i.e. quantitative trait loci (QTL) that are associated with resistance to JD. Although these results are not all consistent (probably due to differences in the study designs, e.g., the population used, the definition of the resistance phenotype, the disease prevalence, or the detection power), overall, they suggest that genetics may help to control the disease. However, apart from a very recent study that estimated the accuracy of genomic predictions [9], little effort has been made to investigate the possibility of predicting resistance to JD from genomic data.

In an earlier study by our group, we conducted GWAS on imputed whole-genome sequences of 1644 Holstein cows with an accurately defined status for MAP (repeated serum ELISA and fecal PCR tests); in this population, we identified various candidate SNPs that accounted for a substantial part of the phenotypic variance in resistance to MAP infection [10]. Using these data, together with additional data collected in infected herds, and a single-step genomic best linear unbiased prediction (GBLUP) model, we conducted a study in Holstein cattle with a two-fold objective: (1) to further investigate the genetic determinism of resistance to JD, and (2) to estimate genetic trends, reliability, and risk factors associated with genomic predictions.

## Methods

### Animals and phenotypes

In this study, we did not perform any experiments on animals; thus, no ethical approval was required. Data from Holstein cows were collected from herds in northwestern France that were enrolled in JD control plans. Two datasets were merged and analyzed. The first dataset was obtained from the PARADIGM project, which contained reliable MAP statuses that were deduced from repeated and concordant serum ELISA and fecal PCR results for 4100 cows. More details can be found in Sanchez et al. [10]. The second dataset, which constituted the majority of the overall dataset, included MAP statuses of 243,274 cows that were deduced from serological tests that have been routinely recorded since 2015. Cows for which all the tests were negative were considered non-infected while cows with at least one positive test were considered infected. Of the entire dataset of 247,374 cows, about 92% were non-infected (n = 228,337) and about 8% were infected (n = 19,037) by MAP. These cows originated from 15,476 herds, of which 5637 had at least one infected cow. All serum samples were analyzed with one of two ELISA kits: Idexx Paratuberculosis Screening Ab Test (Idexx, Montpellier, France) for ~ 75% of the cows and Idvet ID Screen^®^ Paratuberculosis Indirect (Idvet, Montpellier, France) for ~ 25% of the cows.

The PARADIGM dataset had already been cleaned and filtered; on the second dataset, we applied filters as follows. The S/P threshold values (sample optical density over positive control optical density) recommended by the manufacturer were used to distinguish between infected and non-infected cows. However, cows presenting intermediate S/P values, i.e., between 10 and 50, were considered to be of uncertain status and were excluded from the analyses (17.6%). We kept only the cows that were more than 24 months old and that had at least one calving. We also excluded non-infected cows that were less than 3 years old to eliminate individuals that could potentially still be in the latency period. After applying these filters, 119,992 non-infected cows and 18,025 infected cows remained in the dataset. Then, to maximize the probability of exposure to MAP and to estimate the effect of the contemporary group, we kept only birth herd × birth-year combinations with at least one infected and one non-infected cow. We considered the birth herd regardless of the herd in which the animals were located on the day of the test. These filters resulted in a drastically reduced dataset, which ultimately contained 56,766 Holstein cows from 3114 herds. Of these cows, 42,829 were non-infected (~ 75%) and 13,937 were infected (~ 25%).

The pedigree was traced over four generations and contained 161,253 animals, including 12,431 genotyped individuals among which, 4031 cows had MAP statuses (2787 non-infected and 1244 infected). The 12,431 genotyped animals had previously been genotyped with different versions of the 50K SNP Beadchip, including the EuroGMD Beadchip currently used for genomic selection (https://www.eurogenomics.com/actualites/the-eurog-md:-a-unique-genotyping-microarray-for-cattle-.html). In total, 53,469 SNPs passed all quality control filters (individual call rate > 95%; SNP call rate > 90%; minor allele frequency (MAF) > 1%; genotype frequencies in Hardy–Weinberg equilibrium with P > 10^−4^). Missing genotypes were imputed using the FImpute software [11] as part of the French routine system of evaluation (covering more than one million French Holstein animals with genotypes), with a reference population comprising about 35,000 genotyped bulls used for artificial insemination.

### Model

Resistance to JD was modeled as follows:

1 $$\mathbf{y}=\mathbf{X}{\varvec{\upbeta}}+\mathbf{Z}\mathbf{a}+\mathbf{e},$$

where $\mathbf{y}$ is the vector of phenotypes, i.e., 1 for non-infected cows and 0 for infected cows, ${\mathbf{a}}\sim N({\bf{0}},{\mathbf{H}}{{\upsigma }_{\rm{a}}^{2}})$ is the vector of random additive genetic effects, and ${\mathbf{e}}\sim N({\bf{0}},{{\mathbf{I}}}{\upsigma }_{\rm{e}}^{2})$ is the vector of random residual effects. ${\varvec{\upbeta}}$ is the vector of fixed effects of contemporary group (herd of birth × birth year combination), season (birth month), and ELISA test (Idexx or Idvet), to account for variability in exposure to MAP among farms and in time, as well as for differences in test sensitivities and specificities. $\mathbf{X}$ and $\mathbf{Z}$ are incidence matrices, $\mathbf{H}$ is the relationship matrix among individuals that combines genotypic and pedigree information, and $\mathbf{I}$ is the identity matrix, with ${\upsigma }_{a}^{2}$ and ${\upsigma }_{e}^{2}$ as the additive genetic and residual variances, respectively. Variance components were estimated with the WOMBAT software using the AI-REML algorithm [12] and Model (1). The heritability ${h}^{2}$ of resistance to JD was calculated as follows: ${h}^{2}={\upsigma }_{a}^{2}/\left({\upsigma }_{a}^{2}+{\upsigma }_{e}^{2}\right)$.

### Breeding values

Model (1) was applied to the hybrid single-step (HSS) model, proposed by Fernando et al. [13], and implemented in the HSSGBLUP software that is used in the French bovine evaluation. We defined six unknown parent groups depending on the birth year of the animals (≤ 2008, 2009–2010, 2011–2012, 2013–2014, 2015–2016, and 2017–2018). Coherence between pedigree and genotypes was obtained by fitting a $\mathbf{J}$ vector [14, 15] for each unknown parent group. This method directly provides estimates of SNP effects and single-step estimated breeding values (ssEBV) of non-genotyped animals. The ssEBV of the genotyped animals were obtained from genotypes and SNP effects. ssEBV were expressed in genetic standard deviation (${\upsigma }_{a}$) units by dividing predictions obtained on the raw scale by the genetic standard deviation estimated with Model (1) and were centered around a base population consisting of the genotyped cows born from 2015 to 2018.

### Genetic trends

Genetic trends in resistance to JD were assessed separately in females and in males by averaging ssEBV per year of birth. We retained only the animals with the most-accurate ssEBV, i.e., cows with phenotypes (2356 to 8674 cows born each year between 2008 and 2017) and bulls with at least 10 daughters with MAP statuses (31 to 117 bulls born each year between 2000 and 2014). The Interbull method 2 proposed by Boichard et al. [16] was used to test for the absence of bias in the estimate of genetic trends in the bull population. In this approach, the unbiasedness hypothesis is accepted if no significant within-bull birth-year effect is detected on the performance of the daughters after adjustments for environmental effects and the breeding value of the dam estimated by the model. We also calculated within-year correlations between the total merit index and genomic predictions (GP) of resistance to JD obtained after applying the SNP effects previously estimated on 9800 French Holstein bull genotypes.

### Reliability of genomic predictions

Model (1) was applied to a truncated dataset (training) that included the phenotypes of the 41,774 cows born before 2015 (31,115 non-infected and 10,659 infected), of which 3118 had genotypes (2069 non-infected and 1049 infected). The reliability of ssEBV for the animals without phenotypes or progeny was estimated in a validation (VAL) population of 907 cows born between 2015 and 2018 that had both genotypes and MAP statuses (715 non-infected and 192 infected cows). Reliability was estimated by ${r}^{2}/{h}^{2}$, where $r$ is the correlation in the VAL population between MAP status (1 or 0, adjusted for non-genetic effects estimated with Model (1) on the complete dataset) and GP (calculated using SNP effects estimated from the training dataset).

### Relative risk of MAP infection

To assess the risk of MAP infection with respect to GP, we calculated the relative risk (RR) of infection for a cow in the VAL set. A logistic regression of the status (0/1) was carried out with GP as predictor. As an illustration and to check the quality of the fit of the data, these risk factors were also compared to those computed by the ratio of infected to healthy animals in each 0.5 class of GEBV, expressed relatively to class 0.

### Single-step GWAS

To detect QTL for resistance to JD, we performed a single-step GWAS (ssGWAS) [17, 18] from the SNP effects estimated with the HSSGBLUP software. We split the genome into non-overlapping, adjacent 1-Mb regions and calculated the percentage of ssEBV variance ($\%{GV}_{i}$) accounted for by the $i$th genomic region as:

2 $${\mathrm{\%}GV}_{i}= \frac{1}{n{\sigma }_{\alpha }^{2}} {\widehat{\mathbf{a}}}_{i}^{\mathbf{^{\prime}}}{\widehat{\mathbf{a}}}_{i}$$

where $n$ is the sample size, ${\sigma }_{\alpha }^{2}$ is the variance in ssEBV, and ${\widehat{\mathbf{a}}}_{i}={\mathbf{M}}_{i}{\widehat{\mathbf{s}}}_{i}$ is the vector of genomic values for the $i$th region with ${\mathbf{M}}_{i}$ the matrix of SNP content in region $i$ and ${\widehat{\mathbf{s}}}_{i}$ is the vector of estimated effects of the SNPs in the region. We also applied the same approach to whole chromosomes to obtain the ${{\%GV}_{c}}$ explained by each chromosome. Genomic regions with $\%GV$ > 0.05% were annotated with FAANGMine v1.1 (https://faangmine.elsiklab.missouri.edu), which was developed by the Functional Annotation of ANimal Genomes initiative [19] and integrates the ARS-UCD1.2 bovine reference genome with a variety of external data sources, including RefSeq from NCBI (https://www.ncbi.nlm.nih.gov) and Ensembl (https://www.ensembl.org) gene sets.

## Results

### Heritability estimate for resistance to JD

Estimates of genetic and residual variances were 0.019 and 0.113, respectively, corresponding to a moderate ${h}^{2}$ estimate of 0.143 ± 0.01 for resistance to JD. Therefore, the estimate of the genetic standard deviation (used to standardize ssEBV) was 0.019^0.5^, i.e., close to 0.14.

### Genetic trends in resistance to JD

**Fig. 1:**
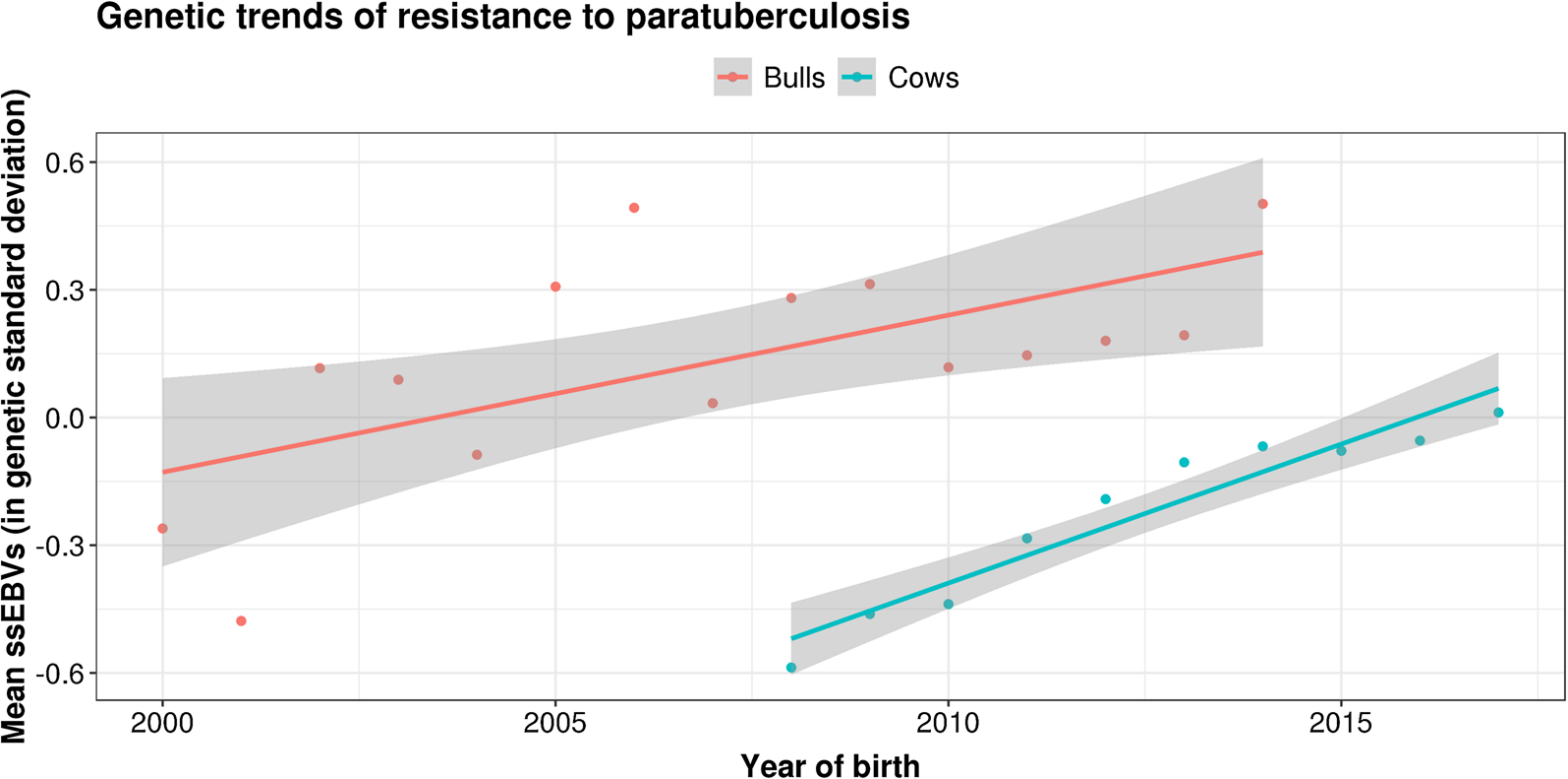
Genetic trends in resistance to paratuberculosis

First, we performed single-step analyses to obtain ssEBV by considering phenotypes and/or genotypes for all the individuals in the pedigree. Genetic trends in the Holstein population were then estimated by averaging ssEBV of cows and bulls by year of birth. In both the bull and cow populations, we observed steadily increasing genetic trends in resistance to JD over the period considered (Fig. 1). In other words, the younger animals appeared to be genetically more resistant than the older ones. Bulls born in 2014 had, on average, ssEBV that were + 0.76σ_*a*_ higher than bulls born in 2000, corresponding to an increase of 0.054${\upsigma }_{a}$ per year. Over a shorter and more recent period (2008–2017), the increase in ssEBV was + 0.60${\upsigma }_{a}$ in cows, i.e., + 0.067${\upsigma }_{a}$ per year. Interbull method 2 did not detect any bias in these estimates of genetic trends. In addition, we obtained a low, but positive (+ 0.061) and favorable, within-birth-year correlation between GP of resistance to JD and the total merit index.

### Reliability of genomic predictions

The effects of SNPs were estimated using a reduced training population and were then applied to the genotypes of the VAL population to predict resistance to JD (GP). The correlation between GP and phenotypes (adjusted for fixed effects) in the VAL group was equal to 0.28 (SE = 0.03). The estimate of genomic reliability, which was obtained by dividing the squared correlation by the value of ${h}^{2}$ that we estimated in the present study, was ~ 0.55.

### Relative risk

**Fig. 2:**
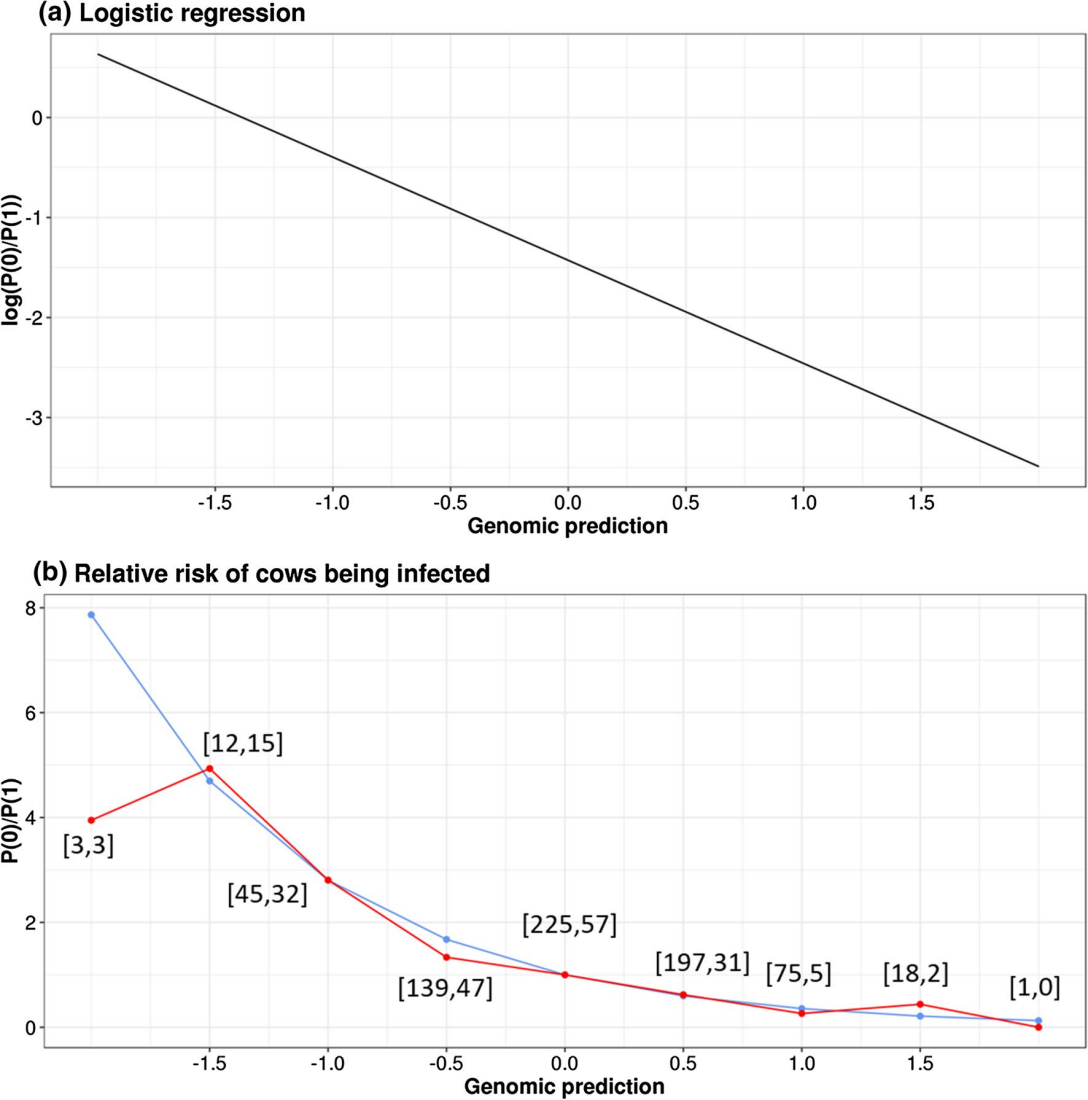
Relative risk of MAP infection in the validation population. **a** Results of the logistic regression (log(*P*(0)/*P*(1)) = − 1.428 to 1.0313 GP) with *P*(0) = probability of cows being infected, *P*(1) = probability of cows being non-infected, and GP = genomic predictions. **b** Relative risks of cows being infected (*P*(0)/*P*(1)) as predicted by logistic regression (in blue) observed within each 0.5 class of genomic predictions (in red). Numbers in parentheses are numbers of non-infected and infected cows, respectively

To assess the effect of genetics on the phenotype, we used the VAL population to estimate the relative risk of cows being infected based on their GP by logistic regression. The regression equation was log(*P*(0)/*P*(1)) = − 1.428–1.0313 GP (*P* < 0.0001). The estimated effect of one genetic standard deviation (− 1.0313) corresponded to a relative risk multiplied by exp(1.0313) = 2.80 when GP decreased by 1, or equivalently a relative risk multiplied by exp(− 1.0313) = 0.36, or divided by 2.8, when GP increased by 1. To illustrate this result, the relative risk, on the log scale and the observed scale, is represented in Fig. 2. Figure 2 also presents the observed relative risk computed within each 0.5 class of GP. Both are quite consistent provided that the number of observations per class is not too small. Combined with the fact that infection occurs early in life, these results show that selection can be an efficient lever to control paratuberculosis.

### Identification of QTL

**Fig. 3:**
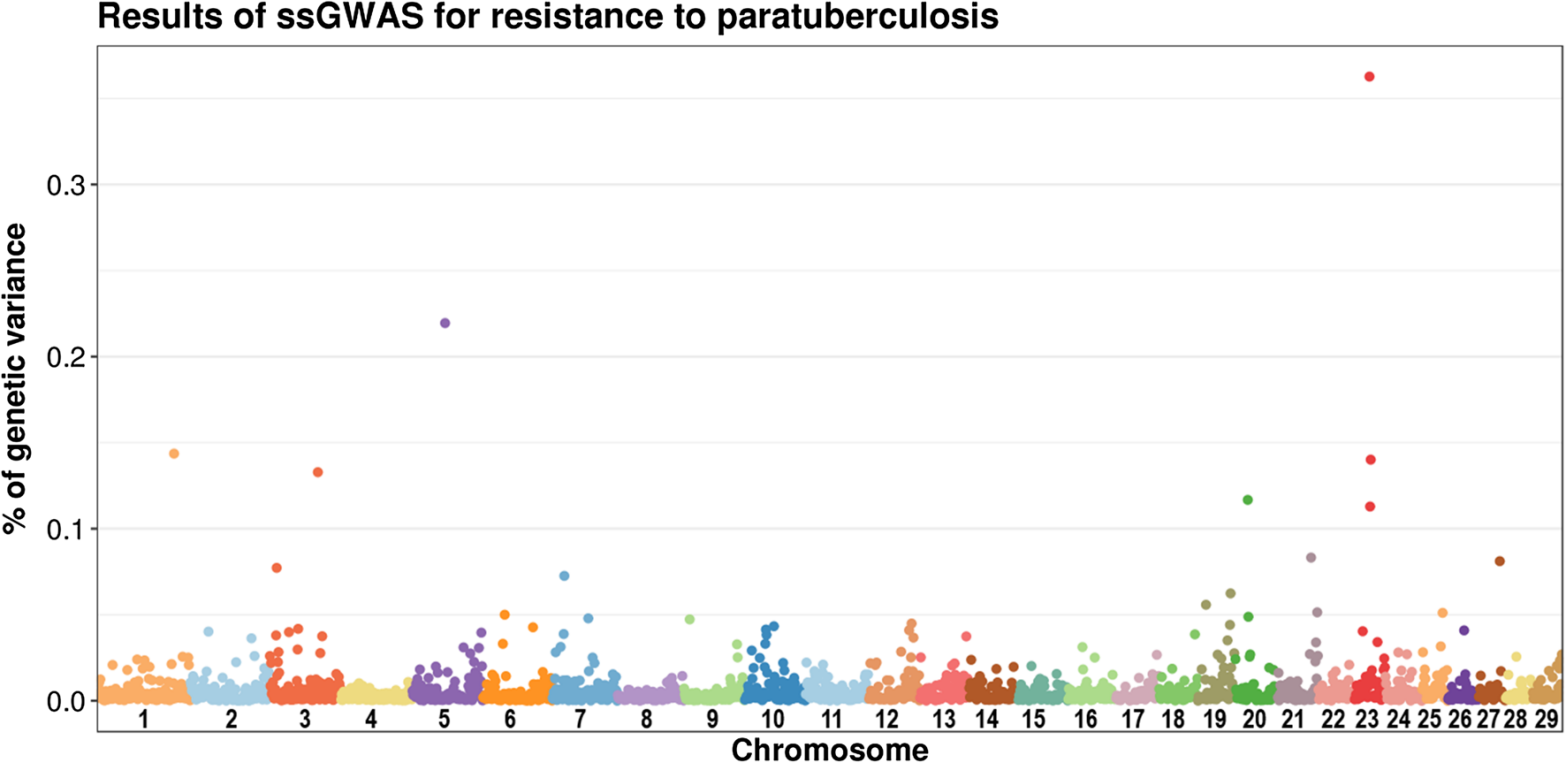
Resistance to paratuberculosis: percentage of ssEBV variance explained by 1-Mb genomic regions. The 29 bovine autosomes are represented by different colors

To identify QTL, i.e., genomic regions with the largest contributions to the genetic variance of the trait, we split the genome into non-overlapping 1-Mb segments, each containing on average 22.5 SNPs (min = 4; max = 199). Then, we calculated the GV percentage in resistance to JD that was explained by each segment, as described in “Methods” section. The 1-Mb genomic regions accounted for 0 to 0.36% GV in this trait. The segment that accounted for the highest GV percentage was located on chromosome 23 and started at 25,207,610 bp (Fig. 3 and Table 1). We also identified lower peaks, which explained 0.05 to 0.22% of the total GV, that were located in other genomic regions on chromosomes 1, 3 (two regions), 5, 6, 7, 19 (two regions), 20, 21 (two regions), 23 (two regions), 25, and 27.

**Table 1:** One-Mb genomic regions that explained more than 0.05% of ssEBV variance in resistance to JD

Chromosome	% ssEBV variance	Start (bp)	Number of SNPs
1	0.144	131,181,000	85
3	0.077	11,556,988	30
3	0.133	82,176,437	45
5	0.219	55,871,314	105
6	0.050	37,030,480	55
7	0.073	20,940,338	25
19	0.056	14,338,858	27
19	0.062	55,308,916	110
20	0.117	21,307,568	117
21	0.083	56,929,149	106
21	0.051	67,739,884	21
23	0.363	25,207,610	52
23	0.113	26,549,915	42
23	0.140	27,565,479	63
25	0.051	33,768,908	57
27	0.081	35,824,524	34

Then, we summed the GV percentages explained by all the 1-Mb regions of each chromosome. The chromosomes with the largest contributions were chromosomes 3 and 23 (1% for each), 5 (0.86%), 1 (0.84%), 7 (0.72%), 10 (0.65%), 2 (0.62%), 12 (0.57%), 19 (0.55%), and 11 and 6 (0.51% each). Other chromosomes accounted for a smaller part of the ssEBV variance, i.e., from 0.18% (chromosome 28) to 0.48% (chromosomes 20 and 21). For the whole genome, 2385 regions were defined that together accounted for 13.6% of the GV in resistance to JD (Table 2). It should be noted that the accumulation of 1 Mb—segment contributions over a chromosome does not take potential covariance between segments into account, which explains this relatively small cumulative contribution. When the same approach to compute contributions was extended to the whole chromosome, we obtained quite different results (Table 2): the most important chromosomes being chromosomes 3 (5.3%), 10 (4.8%), 23 (4.7%), 1 (3.6%), 7 (3.4%), 5 (2.9%), 12 (2.5%), 2 (2.5%), 11 (2.2%), and 13 (2%). Other chromosomes accounted for 0.6% (chromosome 28) to 1.8% (chromosome 21) of the ssEBV variance. Overall, the 29 autosomes accounted for ~ 58% GV.

**Table 2:** Maximal and total percentage of ssEBV variance (% GV) in resistance to JD explained by each autosome

Chromosome	1 Mb non-overlapping regions			Whole chromosome
	Number of regions	Max % GV	Total % GV^a^	% GVc^b^
1	152	0.14	0.84	3.56
2	129	0.04	0.62	2.45
3	116	0.13	1.01	5.33
4	116	0.01	0.31	1.38
5	115	0.22	0.86	2.85
6	113	0.05	0.51	2.22
7	106	0.07	0.72	3.38
8	108	0.01	0.31	1.38
9	99	0.05	0.37	1.49
10	99	0.04	0.65	4.77
11	103	0.02	0.51	2.17
12	83	0.04	0.57	2.50
13	80	0.04	0.44	2.00
14	80	0.02	0.34	1.13
15	81	0.02	0.31	1.11
16	78	0.03	0.33	1.25
17	70	0.03	0.27	1.30
18	63	0.04	0.30	0.77
19	61	0.06	0.55	1.54
20	69	0.12	0.48	1.55
21	66	0.08	0.48	1.80
22	59	0.02	0.30	1.45
23	50	0.36	1.00	4.69
24	60	0.03	0.28	1.05
25	42	0.05	0.39	1.56
26	50	0.04	0.20	0.71
27	44	0.08	0.25	0.88
28	44	0.03	0.18	0.60
29	49	0.03	0.27	1.05

^a^By summing the % GV for all the 1-Mb regions of each chromosome

^b^By considering each chromosome as a single region

## Discussion

This study, conducted in Holstein cows, is based on a single-step SNP-BLUP evaluation using MAP statuses that were deduced from serological data, which are routinely recorded in French herds enrolled in paratuberculosis control plans. It provides new insights into the genetic determinism of resistance to MAP and gives very encouraging results for the implementation of this kind of genomic evaluation in Holstein cattle.

### New insights into the genetic determinism underlying resistance to JD

To the best of our knowledge, the current study is one of the few studies that estimates the heritability of the resistance to JD from thousands of cows with serum ELISA measurements. The obtained ${h}^{2}$ value is moderate (0.14), but similar to or higher than estimates that have been reported in the literature (from 0.04 to 0.18) from serum ELISA phenotypes measured in various Holstein populations [20–24]. However, our value is much lower than the value that we had estimated in a previous study (0.57) from a genomic relationship matrix [10], probably because our earlier study was conducted with a smaller sample (1644 Holstein cows) with both very accurate (confirmed by PCR on feces) and strongly selected phenotypes.

To identify genomic regions that are involved in the genetic determinism underlying resistance to JD, we applied a multi-SNP GWAS approach that analyzed 161,253 animals in the pedigree, including 56,766 with phenotypes and 12,431 with genotypes. Previous studies have demonstrated equivalence between GBLUP and classical single-marker GWAS, e.g., [25], and furthermore, it has been shown that the detection of QTL via GBLUP can be extended to single-step GBLUP, also referred to as ssGWAS [17, 18]. In recent years, ssGWAS approaches, which have the advantage of estimating all SNP effects jointly, have been applied in multiple studies to identify QTL for different traits, e.g., [26]. When applied to resistance to JD in our Holstein cattle dataset, it highlighted regions of the genome located on chromosomes 1, 3, 5, 6, 7, 19, 20, 21, 23, 25, and 27, which account for the largest GV percentage in this trait (> 0.05%). However, this method has some limitations. First, it requires genomic regions to be defined arbitrarily (e.g., fixed size in bp or fixed number of SNPs, overlapping or non-overlapping intervals), which can have an impact on the results, in particular if the density of SNPs in the genome varies from one region to another. In the current study, we tested intervals of different sizes and with different numbers of SNPs that overlapped or not, and obtained comparable results in all cases (data not shown). In addition, we found that when we added up the GV percentages in resistance to JD across all the 1-Mb genomic regions, only slightly more than one-eighth of the total GV in the trait was accounted for. This result is likely due to the long-range linkage disequilibrium (LD) that occurs in the genome of dairy cattle and the distribution of QTL effects over a large number of potentially distant markers. To test this hypothesis, we calculated the percentage of ssEBV variance explained by each chromosome as a whole (i.e., by considering each chromosome as a single segment), and found that the total GV percentage explained by the whole genome was four times higher (58%) than when we considered the 1-Mb genomic regions. This result supports our hypothesis about the impact of long-range LD and also seems to provide evidence for a notable degree of covariance between SNPs on different chromosomes, supporting the existence of LD between SNPs located on different chromosomes [27].

Consistent with our earlier study of imputed whole-genome sequences from 1644 Holstein cows with extreme phenotypes [10], our findings confirm the effects of genomic regions located on chromosome 23. However, there are some notable differences between the two analyses. Our current study reveals new regions that affect resistance to JD that were not identified in our earlier work, and fails to confirm the major effects that we had previously detected, at the sequence level in a single-SNP GWAS, on chromosomes 12 and 13 [10]. It is noteworthy that, in these two QTL regions detected at the sequence level, variants were rare and probably in low LD with the 50k SNPs, especially in this much larger population. In spite of this, in the whole-chromosome analysis presented here, chromosomes 12 and 13 accounted for 2.5 and 2% of the ssEBV variance in resistance to JD, which ranks them 7th and 11th out of the 29 autosomes, respectively. This discrepancy is not very surprising given that there has been little agreement among the numerous GWAS analyses that have been conducted to date (using SNPs at different densities) for resistance to JD. The genomic region located in the vicinity of the major histocompatibility complex on chromosome 23 is the only QTL that has been detected by almost all published GWAS, in particular the most recent ones that were performed with high-density SNP genotypes or imputed whole-genome sequences [8, 9, 28, 29]. Most of the other genomic regions detected in this study were previously described as affecting resistance to JD in at least one GWAS. These regions are located on chromosomes 1 [7, 30], 3 [8, 28], 7 [28, 31], 20 [28, 32], 21 [8, 32], 25 [8], and 27 [28, 33]. In contrast, the genomic regions that we identified on chromosome 5 at ~ 56 Mb, on chromosome 6 at ~ 37 Mb, and on chromosome 19 at ~ 14 and ~ 55 Mb, have not, as far as we know, ever been identified. In these regions, we identified a number of positional candidate genes, among which, seven are plausible functional candidates. The *ATG4D* gene on chromosome 3 is an autophagy-related gene. Autophagy is a cellular process that can eliminate intracellular pathogens, including bacteria. Moreover, the *ATGD4* gene is known to be associated with resistance to bovine paratuberculosis [9] and human Crohn’s disease [34], which is a chronic inflammatory bowel disease with a possible link to paratuberculosis. Silencing of the *LRP1* gene, located in the QTL region on chromosome 5, has been found to exacerbate inflammatory response in mice [35]. The *GNG7* and *GADD45B* genes on chromosome 7 were both found to be differentially expressed in ileal lymph nodes of MAP-infected and non-infected cows [36] and in MAP-infected bovine monocyte-macrophages and uninfected bovine cells [37], respectively. The *BOLA-DRB3* gene, which is located in the major histocompatibility complex on chromosome 23, has been shown to be associated to bovine paratuberculosis in different studies (see review by Kravitz et al. [38]). The *HIP1* gene (chromosome 25) plays a role in the susceptibility to tuberculosis, which is an infection that is very similar to MAP at the cellular level. *HIP1* promotes pathogenesis, by impairing host immune responses [39]. Finally, the *ANK1* gene on chromosome 27 has been described as an up-regulated gene in MAP-infected mice compared to control mice [40]. Causality of these genes is yet to be demonstrated, but they are very good functional candidates to explain the effects that we observed in the current study.

### Resistance to JD can be predicted from SNP genotypes

In a validation population, we obtained relatively reliable genomic predictions for resistance to JD (reliability = 0.55), which were estimated from a reduced reference population that included about 75% of cows with phenotypes. Such genomic predictions can be of great help to identify cows at high risk of infection. As mentioned in the “Background” section, only one prior study has estimated the accuracy of genomic predictions for resistance to JD using cross-validation [9]. Those authors estimated correlations between genomic predictions for bulls and daughter averages for MAP infection, and obtained values ranging from 0.43 to 0.53. Although these correlation values are higher than those estimated here, the daughter averages used in that study are likely to be more heritable than the cows’ individual performances; if this is taken into account, the reliability value is probably on the same order of magnitude as that found in our study (0.55).

When estimated over the last two decades, trends in genomic predictions reveal a favorable genetic evolution in resistance to JD. These trends are supported by a method recommended by Interbull. Indeed, no significant within-bull birth year effect was observed on the phenotypes of the daughters adjusted for fixed effects and breeding value of the dam. We also found a slightly positive correlation between the total merit index, produced by the national genetic evaluation of the French Holstein population, and the breeding values estimated for resistance to JD, suggesting that the favorable genetic trend observed for resistance to paratuberculosis could be the result of indirect selection in the Holstein breed.

Currently, breeding values for resistance to paratuberculosis are routinely produced. Such values can be used for two important objectives: (i) in infected herds, this information is useful for management decisions, to guide the early culling of highly susceptible animals and plan matings with resistant bulls; and (ii) at the level of the overall breeding scheme, these breeding values can be integrated into the breeding goal and, more importantly, used to produce bulls for artificial insemination that are resistant to JD and can be used in infected herds.

As the number of genotyped and phenotyped cows increases, genomic predictions will rapidly become an effective tool for controlling paratuberculosis on French Holstein farms. The same strategy is currently being developed in the Normande breed and could be extended to other populations in the future.

## Conclusions

Using a single-step genomic evaluation applied to a large dataset of Holstein cows, we confirm the genetic determinism that underlies host resistance to paratuberculosis. Overall, the heritability estimate for resistance to this disease was 0.14 and we identified various regions on the genome that account for a substantial part of the ssEBV variance in resistance to paratuberculosis in Holsteins, with the largest contributions due to regions located on chromosomes 23, 5, 1, and 3. In these regions, association analyses at the sequence level allowed the identification of individual genes and gene variants that are responsible for resistance to paratuberculosis. Genomic predictions for this trait were found to have a satisfactory reliability (0.55), which means that such predictions could be of great interest to identify cows at high risk of infection and to highlight resistant bulls for inclusion in mating plans. This approach could be easily applied in any breed of cattle affected by this disease for which a similar number (i.e., a few thousand) of cow phenotypes and genotypes are available.

## Data Availability

These data (genotypes and phenotypes) are part of a reference population used for genomic selection and have commercial value. Therefore, restrictions apply to their availability and they are not publicly available. The authors can be contacted for a reasonable request.
